# MyoD Is a Novel Activator of Porcine *FIT1* Gene by Interacting with the Canonical E-Box Element during Myogenesis

**DOI:** 10.3390/ijms161025014

**Published:** 2015-10-20

**Authors:** Chi Yan, Xiaoliang Xia, Junxian He, Zhuqing Ren, Dequan Xu, Yuanzhu Xiong, Bo Zuo

**Affiliations:** 1 Key Laboratory of Swine Genetics and Breeding of the Ministry of Agriculture, College of Animal Sciences & Technology, Huazhong Agricultural University, Wuhan 430070, China; E-Mails: yanchi_2005@126.com (C.Y.); xxl8365@163.com (X.X.); renzq@mail.hzau.edu.cn (Z.R.); dequanxu@mail.hzau.edu.cn (D.X.); 2 Yuguan Agricultural Inc., Shuining 629208, China; E-Mail: hejunxian2011@yahoo.com

**Keywords:** porcine *FIT1* gene regulation, C2C12 myotube, E-box, MyoD1, ChIP, muscle

## Abstract

Fat-induced transcript 1 (*FIT1*/*FITM1*) gene is a member of the conserved gene family important for triglyceride-rich lipid droplet accumulation. *FIT1* gene displays a similar muscle-specific expression across pigs, mice, and humans. Thus pigs can act as a useful model of many human diseases resulting from misexpression of *FIT1* gene. Triglyceride content in skeletal muscle plays a key role in pork meat quality and flavors. An insertion/deletion mutation in porcine *FIT1* coding region shows a high correlation with a series of fat traits. To gain better knowledge of the potential role of *FIT1* gene in human diseases and the correlations with pork meat quality, our attention is given to the region upstream of the porcine *FIT1* coding sequence. We cloned ~1 kb of the 5′-flanking region of porcine *FIT1* gene to define the role of this sequence in modulating the myogenic expression. A canonical E-box element that activated porcine *FIT1* promoter activity during myogenesis was identified. Further analysis demonstrated that promoter activity was induced by overexpression of MyoD1, which bound to this canonical E-box during C2C12 differentiation. This is the first evidence that FIT1 as the direct novel target of MyoD is involved in muscle development.

## 1. Introduction

Fat-induced transcript 1 (*FIT1/FITM1*), also named fat-inducing transcript 1, is a member of the conserved gene family (including *FIT1* and *FIT2*), important for triglyceride-rich lipid droplet (LD) accumulation [1]. Intramuscular triglyceride content plays a key role in determining pork flavor [2]. Li *et al.* [3] showed that an insertion/deletion mutation in porcine *FIT1* gene coding region was highly associated with a series of fat traits that significantly affect pork meat quality. As shown previously in our lab, porcine *FIT1* exhibited a particular and high expression in skeletal muscle tissues with extremely low expression in fat tissue [4]. Mouse FIT1 mRNA was detected at a high level in heart and skeletal muscles while FIT1 protein was detected mainly in skeletal muscles and was relatively lower in the heart. Human FIT1 mRNA and protein showed a similar expression pattern with mice and pigs [1,4], suggesting the conserved role of *FIT1* in regulating myogenic expression among these mammals. In addition, very limited reports [5] showed that *FIT1* gene was found differentially expressed in muscles from normal individuals and facioscapulohumeral muscular dystrophy (FSHD) patients. Collectively, study on the transcriptional regulatory mechanism of porcine *FIT1* gene is of high agricultural and medical value.

Previous investigation into *FIT1* gene mainly focus on its involvement in triglyceride storage, and little information is available to elucidate the role in detail in muscle development apart from the limited knowledge on expression profiling. As *FIT2* was primarily expressed in adipose tissue [1], functions of FIT proteins on fat accumulation tended to be put together. Specifically, murine FIT protein were both localized in endoplasmic reticulum (ER) with six-transmembrane-domains each, and mediated triglyceride-rich LD accumulation by direct binding to each protein [1,6,7]. However, this difference in expression pattern may reflect their individual unique function [7]. Our experiments on *FIT1* gene regulation in skeletal muscle in pigs extend this work and show the unique function of FIT1 in muscle.

In this study, we characterized the proximal *FIT1* promoter and identified a canonical E-box element that activated the promoter activity during differentiation. We further demonstrated that the activity of *FIT1* promoter could be induced by MyoD1 which bound to this conserved E-box site.

## 2. Results

### 2.1. FIT1 Expression in the Developing Skeletal Muscle and the Differentiated C2C12 Cells

Previous reports showed that the transcript of porcine *FIT1* was mainly detected in skeletal muscle and heart tissues. To further investigate whether *FIT1* gene expression was regulated in a temporal manner during skeletal muscle development of swine, the expression of FIT1 mRNA was examined at various developmental stages, including embryonic, 2-, 4-, 6-month of age respectively (Figure S1A). Results showed porcine FIT1 was expressed at the highest level at 4 months old (~4 folds, *p* < 0.01) with the lowest level at the embryonic stage while the level dropped dramatically (~2 folds, *p* < 0.05) at 6 months old. This indicates that *FIT1* expression is regulated with development of skeletal muscles in pigs.

To further determine whether the expression of *FIT1* gene in C2C12 myoblasts was regulated in a similar way, FIT1 mRNA was quantitated using myoblast cultures and myotubes differentiated for 2, 4, and 6 days, respectively. As shown in Figure S1B, the expression level was extremely low at the undifferentiated state. At day 2 after differentiation, FIT1 increased rapidly (~15 folds, *p* < 0.001) whereas the expression level was reduced markedly at day 4 (~5 folds, *p* < 0.001). Following differentiation for another two days, though the decrease continued, the expression level of *FIT1* remained higher than that of C2C12 myoblasts (~3.5 folds, *p* < 0.01). The data above demonstrates that the expression of *FIT1* in C2C12 cells is induced with differentiation. In summary, our results illustrated that *FIT1* was temporally expressed in the similar pattern in C2C12 cells as that in skeletal muscle.

### 2.2. Sequence Analysis of the Porcine FIT1 5′-Flanking Region

**Figure 1:**
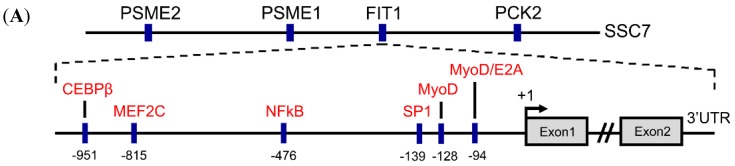

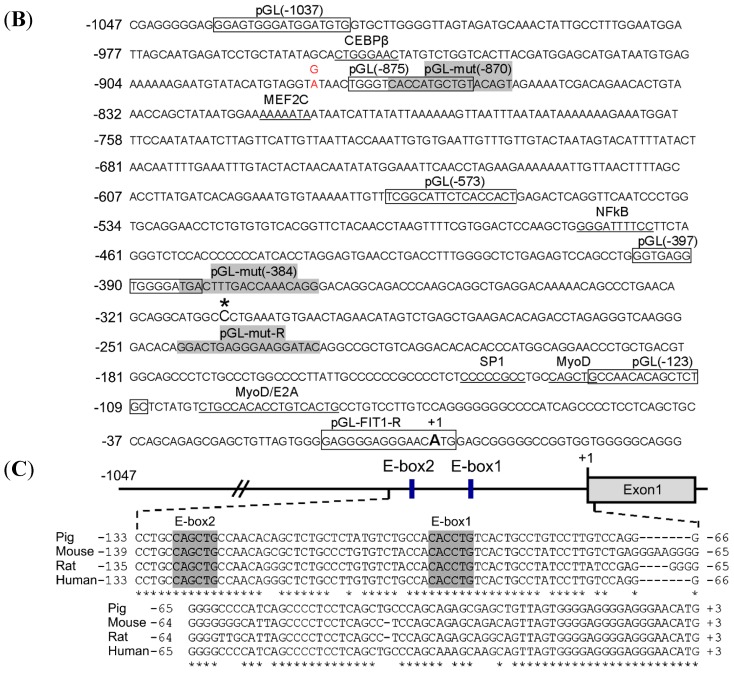
Sequence analysis of the 5′-flanking region of porcine *FIT1* gene. (**A**) Schematic of the region surrounding *FIT1* gene on the chromosome 7 of the pig genome (SSC7) as well as the transcription factor binding sites in the 5′-flanking region of *FIT1* gene. **Top**: blue bars represent gene positions. *PSME2*, proteasome activator complex subunit 2 gene; *PSME1*, proteasome activator complex subunit 1 gene; *PCK2*, phosphoenolpyruvate carboxykinase 2 (mitochondrial) gene; **Bottom**: The transcription factors are indicated in red color, with their individual binding sites in blue bars. The translation initiation site is designated as +1. CEBPβ, CCAAT/enhancer-binding protein β; MEF2C, myocyte-specific enhancer factor 2C; NFκB, nuclear factor κβ; SP1, specificity protein 1; MyoD1, myogenic differentiation 1; E2A, E-protein transcription factors E12 and E47; (**B**) nucleotide sequences of the 5′-flanking region of porcine *FIT1* gene. The transcription initiation site is indicated with an asterisk; putative transcription factor binding sites are underlined with names indicated above the line. The primers for the promoter deletion amplification are boxed and the primers for the mutated constructs are highlighted in gray color. The base in red color denotes the polymorphic site between Large White pig and Meishan pig; (**C**) sequence alignment of the proximal promoter of *FIT1* gene across pig, mouse, rat and human. Two highly conserved MyoD binding sites (E-box1: CACCTG and E-box2: CAGCTG) among these species are indicated in gray color with names above the sequence and their relative positions in the promoter are shown in blue bars. Asterisk: conservative bases across these species.

Approximately 1 kb of the *FIT1* 5′-flanking region (−1037 to +3 bp, relative to the translation initiation site) was obtained using porcine genomic DNA as the template. Using TESS software, several putative regulatory factor-binding sites related to muscle development were predicted, including CEBPβ, NFκB, MyoD, and MEF2C. A graphical summary of transcription factor binding sites of *FIT1* 5′-flanking region was shown (Figure 1A, bottom). *FIT1* gene was localized on the porcine chromosome 7 (LOC00152032) and the adjacent genes of *FIT1* were identified, including *PSME1*, *PSME2* and *PCK2* (Figure 1A, top). The transcription factor binding sites to the porcine *FIT1* 5′-flanking region were identified and the transcription initiation site was located at −310 bp upstream of the ATG start codon (Figure 1B). Alignment of the 5′-flanking regions between Large White pig and Meishan pig showed that a polymorphic site was located at the −880 bp upstream (Figure 1B). In addition, alignment of the proximal sequence of this region among pig, mouse, rat and human indicated that two highly conserved MyoD binding sites (denote E-box1 and E-box2) were present across these species (Figure 1C).

### 2.3. Transcriptional Activation of Porcine FIT1 Gene in Differentiated C2C12 Cells

**Figure 2:**
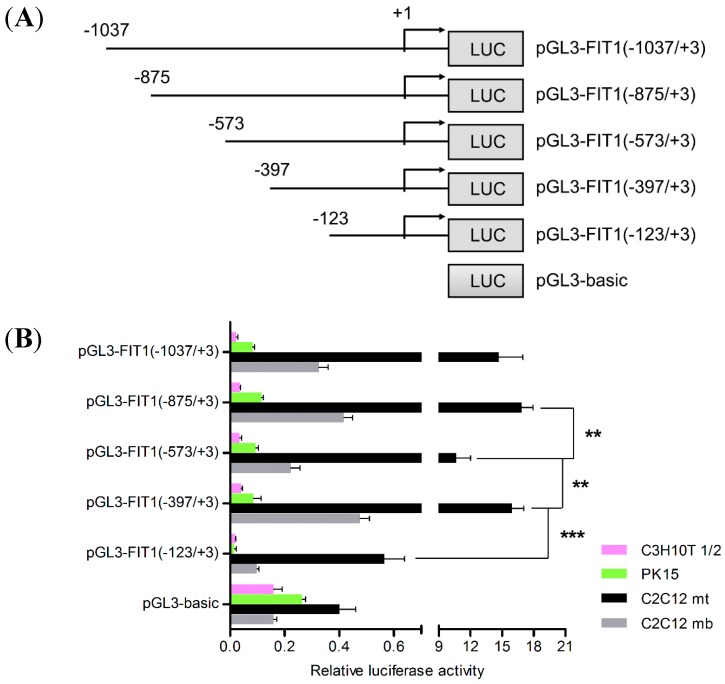

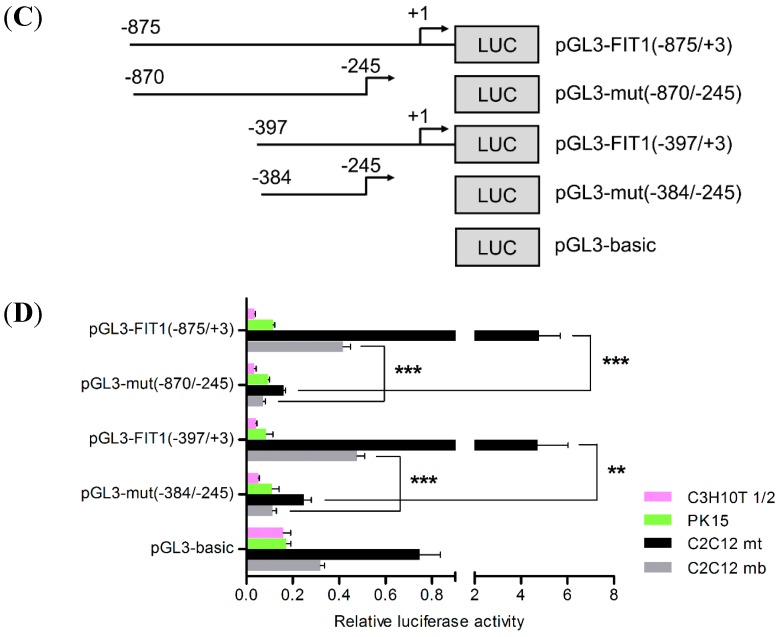
Promoter activities of porcine *FIT1* gene are activated by the conserved E-boxes in C2C12 myotubes. (**A**) Schematic structures of various progressive deletions in the 5′-flanking region of the porcine *FIT1* gene; (**B**) activities of the deletion fragments of the porcine FIT1 promoter in myogenic and non-myogenic cells. Data are shown as the means ± SD (*n* = 4). ** *p* < 0.01; *** *p* < 0.001 by ANOVA. Purple bar denotes C3H10T 1/2 cells; green bar denotes porcine PK15 cells; black bar denotes C2C12 myotubes (mt); gray bar denotes C2C12 myoblasts (mb); (**C**) the graphic indication of the new mutated deletions of the conserved E-boxes from the promoter region; (**D**) activities of the new mutated fragments in myogenic and non-myogenic cells. The fragments of pGL3-FIT1(−875/+3) and pGL3-FIT1(−397/+3) were used as positive control. Data are shown as the means ± SD (*n* = 3). ** *p* < 0.01; *** *p* < 0.001 relative to the positive control (2-tailed student’s *t*-test). Purple bar denotes C3H10T 1/2 cells; green bar denotes porcine PK15 cells; black bar denotes C2C12 myotubes (mt); gray bar denotes C2C12 myoblasts (mb).

To determine whether this 1 kb region had promoter activity, various deletion fragments were introduced to a luciferase reporter vector (Figure 2A). Next, these fragments were delivered into undifferentiated and differentiated C2C12 cells, C3H10T 1/2 fibroblasts, and PK15 cells. Transcriptional levels were assayed using dual luciferase activity assay. Results revealed there was no transcription activity for the longest and deletion fragments in C3H10T 1/2 fibroblast cells, and PK15 cells (Figure 2B). However, the activity of these fragments in C2C12 myoblasts was extremely low (Figure 2B). In contrast, in C2C12 myotubes the fragment between −875 to +3 bp showed the highest luciferase activity (~42 folds above pGL3-basic, *p* < 0.001) whereas the region from −1037 to +3 bp had slightly lower luciferase activity. Results from multiple comparisons showed that the luciferase activity variation was not significant between these two fragments (*p* > 0.05). Moreover, the shorter fragment from −397 to +3 bp displayed higher activity (~94% activity of −875 to +3 bp, Figure 2B), indicating that the region −397 to +3 bp as the core promoter contained essential elements required for myogenic transcription. Through multiple comparisons, significant difference was also found between fragments −875/+3 and −573/+3 in C2C12 myotubes, indicating that MEF2C might serve as a positive regulatory factor (*p* < 0.01, Figure 2B). This data suggests the promoter activity of FIT1 is induced with C2C12 differentiation.

### 2.4. The E-Box Elements in the Core Promoter Are Required for Porcine FIT1 Transcriptional Activation during C2C12 Myogenesis

To further identify the specific elements in the shorter fragment (−397 to +3 bp) of *FIT1* gene responsible for transcriptional activation in C2C12 myotubes, we constructed two more plasmids (−384 to −245 bp and −870 to −245 bp, respectively) excluding the potential E-boxes (Figure 2C) according to the prediction by TESS. The two regions (−875/+3) and (−397/+3) act as positive controls (Figure 2C). Constructs were cotransfected with the internal control pRL-TK into C2C12 myoblast and myotube cultures, C3H10T 1/2 fibroblasts, and PK15 cells using the same method as above. None of transcription activity was observed for new deleted fragments in these cells (Figure 2D). By comparisons, significant differences in luciferase activity were found between fragments (−875/+3) and (−870/−245) (*p* < 0.001) as well as fragments (−397/+3) and (−384/−245) (*p* < 0.01) in C2C12 myotubes, respectively (Figure 2D). This suggests the region (−245 to +3 bp) comprising both E-box elements is necessary for the promoter activity of *FIT1* in C2C12 myotubes, indicating the presence of a potential myogenic enhancer site in this region.

### 2.5. The Canonical E-Box1 Element within Porcine FIT1 Promoter Plays the Dominant Role in this Activation during C2C12 Myogenesis

**Figure 3:**
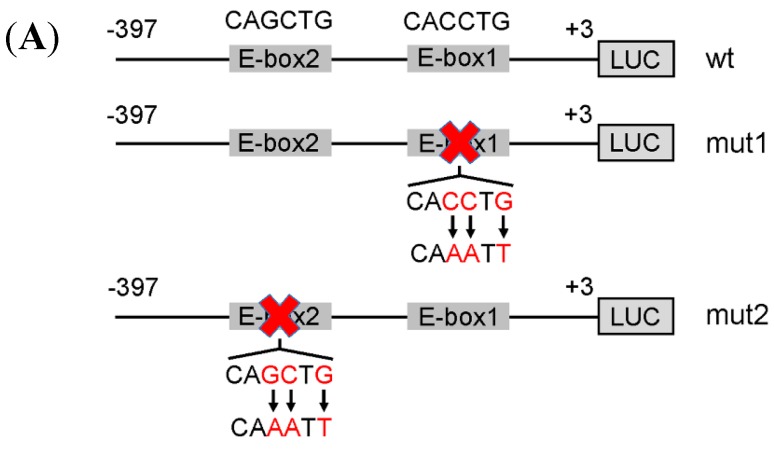

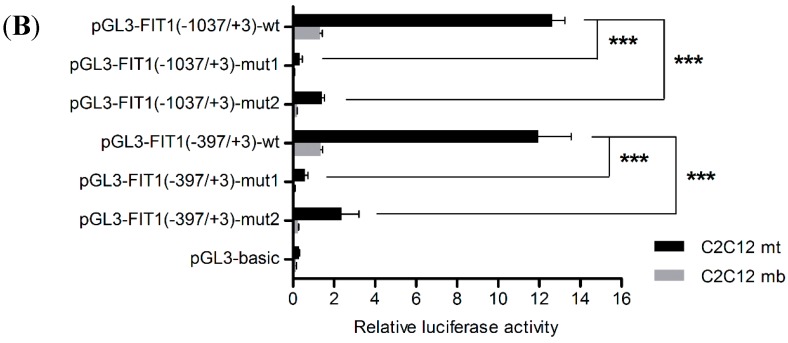
Activities of porcine *FIT1* promoter in which either E-box element was site-directed mutated during C2C12 myogenesis. (**A**) Schematic structures of various site-directed mutants of either E-box site in the fragment −397/+3. mut1 denotes the sequence of the E-box1 site alone mutated from CACCTG to CAAATT and mut2 denotes the sequence of E-box2 site alone mutated from CAGCTG to CAAATT, each indicated with a red check mark, respectively. The mutated bases are indicated in red; (**B**) the activities of wild-type fragments (−397/+3 and −1037/+3, respectively) and different mutants of porcine *FIT1* promoter in undifferentiated and differentiated C2C12 cells. Data are the means ± SD (*n* = 3). *** *p* < 0.001 relative to the values of wild-type fragments (2-tailed student’s *t*-test). Black bar denotes C2C12 myotubes (mt); gray bar denotes C2C12 myoblasts (mb).

To further confirm the role of either E-box sequence in the region −245 to +3 bp of the *FIT1* promoter, we introduced site-directed mutagenesis to E-box1 and E-box2 of the fragments −1037 to +3 bp and −397 to +3 bp respectively (Figure 3A). At the same time, further sequence analysis confirmed that both E-boxes were present and conserved among pig, human, mouse and rat (Figure 1C). Our data showed that mutations in either E-box1 or E-box2 significantly reduced the activity of *FIT1* promoter in these two fragments (*p* < 0.001; Figure 3B). However, it is noted that mutations of E-box1 in both fragments blocked almost the full activity of *FIT1* promoter in differentiated C2C12 cells (>95% activity of each; Figure 3B). This suggests that at least to some extent E-box1 governs the promoter activity and is required for myogenic activation of *FIT1* gene during C2C12 differentiation.

### 2.6. MyoD1 Promotes Porcine FIT1 Transcription during C2C12 Differentiation

**Figure 4:**
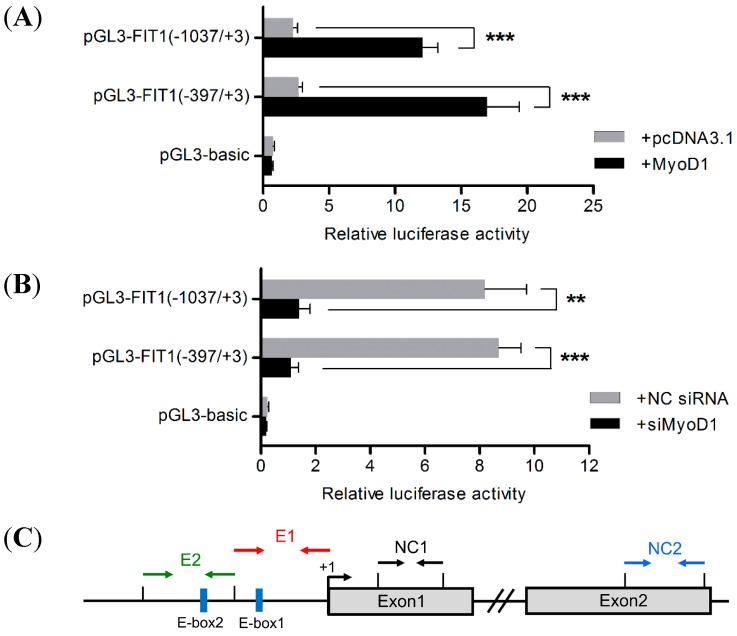

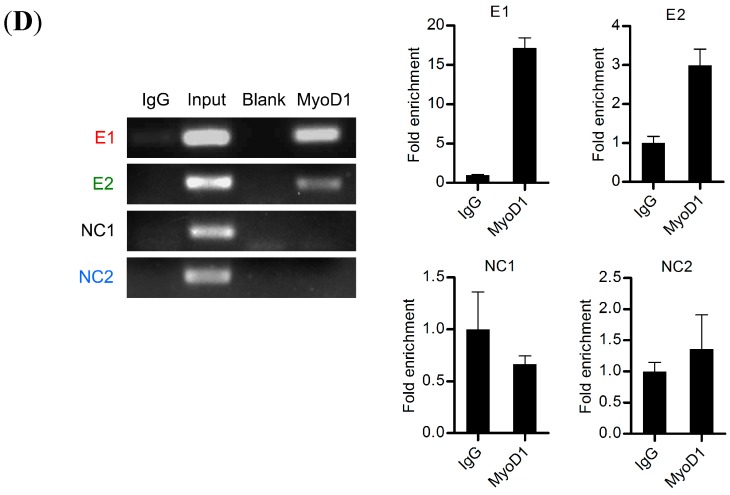
MyoD1 improves the activity of *FIT1* promoter and binds to the canonical E-box1 site during C2C12 myogenesis. (**A**) Overexpression of MyoD1 greatly increased *FIT1* promoter activity in C2C12 myotubes. The pcDNA3.1 plasmid was used as a negative control. Data are the means ± SD (*n* = 3). *** *p* < 0.001 relative to the negative control (2-tailed student’s *t*-test); (**B**) knockdown of MyoD1 by siRNA in C2C12 myotubes dramatically decreased the activity of *FIT1* promoter. The NC siRNA was used as a negative control. Data are shown as the means ± SD (*n* = 3). ** *p* < 0.01; *** *p* < 0.001 relative to the negative control (2-tailed student’s *t*-test). Black bar denotes experiment group; gray bar denotes control group; (**C**) the relative positions of the primers used in ChIP qPCR reactions. The relative positions of each primer pair are indicated in arrows with the same color. Primer sets of E1 and E2 were used to amplify E-box1 and E-box2 regions specific to *FIT1* promoter, respectively. As negative controls, primer sets of NC1 were used to amplify the exon1 region (without E-box); primer sets of NC2 were used to amplify the exon2 region (with one E-box), which was not expected to interact with MyoD1; (**D**) the relative enrichments of either E-box region in *FIT1* promoter by ChIP. Input served as the internal control and data were normalized to IgG by ∆∆*C*t method. Data are indicated with the means ± SD (*n* = 3). A representative image was shown for each primer set. Total chromatin was used as the input. IgG was used as a negative control; blank indicated no template in the PCR reaction.

Evidence demonstrates that myogenic transcription is activated by MyoD-E12/E47 heterodimers which bind to a consensus E-box sequence (CANNTG) in muscle-specific gene promoter or enhancer regions [8]. In combination with our sequence analysis (Figure 1B), we cotransfected with the fragments −1037 to +3 bp and −397 to +3 bp the plasmid harboring the coding sequence of MyoD1 into C2C12 cells to confirm the role of MyoD1 in *FIT1* promoter. Results showed that over-expression of MyoD1 significantly enhanced the promoter activity (~5 folds, *p* < 0.001 and ~6 folds, *p* < 0.001 respectively; Figure 4A). To further prove this, siRNA against MyoD1 was transfected into the C2C12 myotubes with the two regions shown above. Inhibition of MyoD1 during differentiation dramatically decreased the *FIT1* transcription activity (~6 folds, *p* < 0.01 and ~8 folds, *p* < 0.001 respectively; Figure 4B). In sum, these results demonstrate that MyoD1 is crucial to the induction of *FIT1* promoter activity during C2C12 differentiation.

### 2.7. Binding of MyoD1 to the Canonical E-Box1 Element within Porcine FIT1 Promoter in C2C12 Myotubes

As described above, *FIT1* gene displayed a typical characterization of high and primary muscle expression patterns among mammals. In addition, it was shown that there was an enhancer site present in the porcine *FIT1* promoter containing the conserved E-box motifs that were required for myogenic regulation and the promoter activity was induced by MyoD1 overexpression. It was previously reported that MyoD transcription factor bound to the *cis*-element that contained a core consensus sequence specified by CANNTG [9]. To confirm whether this sequence located in the promoter of *FIT1* interacted with MyoD1, we carried out ChIP experiments in C2C12 myotubes using the MyoD1 monoclonal antibody. PCR amplification was performed with immunoprecipitated chromatin samples and qRT-PCR was carried out to determine the enrichment of this region relative to the amount of total input. A schematic of the primers used in ChIP-qPCR was shown in Figure 4C. The results showed that MyoD1 bound to the region of *FIT1* promoter from −123 to +3 bp containing simply E-box1 site (Figure 4D, left panel: E1) and the relative enrichment was ~17 folds over IgG based on three independent experiments (Figure 4D, right panel: E1). In addition, the region containing E-box2 site alone was observed in the *FIT1* promoter as well using site-specific primers (Figure 4C,D, left panel: E2) with ~3 folds enrichment (Figure 4D, right panel: E2); however, for negative controls NC1 and NC2, none of enrichments were detected to interact with MyoD1 using specific primers (Figure 4C,D, NC1, NC2). Thus, we conclude that MyoD1 binds to the canonical E-box1 site and activates the promoter activity of *FIT1* via the interaction with this E-box1 site.

## 3. Discussion

*FIT1* gene plays an important and conserved role in triglyceride-rich lipid droplet formation and has a very high and particular expression in skeletal muscle of pigs, humans and mice [1,4]. We isolated the promoter region of porcine *FIT1* gene and for the first time, identified a myogenic enhancer regulatory element within the promoter in C2C12 myotubes. Our data demonstrated that MyoD1 induced transcription of porcine *FIT1* gene by binding to the conserved E-box1 sequence within this enhancer site. These findings revealed that MyoD as a muscle-specific transcription factor [10], was involved in the transcriptional activation of porcine *FIT1* gene. The pig growingly acts as an animal model for many diseases due to its similarity to humans [11]. It was mentioned in a recent patent that *FIT1* gene was differentially expressed in muscles from normal individuals and FSHD muscular dystrophy patients [5], thus our work may provide some reference for possible involvement of *FIT1* in this muscle disease in future study. Also, we examined the expression level of *FIT1* during various developmental stages of skeletal muscle in domestic pigs. Our results showed that *FIT1* expression was lowest at the embryonic stage and increased after birth, with the maximum at 4 months old in longissimus dorsi muscles of Large White pigs. This suggests the potentially important function of *FIT1* in the postnatal muscular growth and the quality of pork meat. During porcine postnatal development, skeletal muscle was specificated into mature adult type I (slow-oxidative), type IIA (fast oxido-glycolytic), and type IIX and IIB (fast glycolytic) [12]. Research on muscle metabolism of growing pigs indicated that oxidative capacity declines while glycolytic capacity increases with age [13]. In addition, the percentage of type I fibers increased from birth up to 2 months old and little change occurred thereafter [14]. In contrast, glycolytic fibers were detectable only from one month and 4 months old in longissimus dorsi muscles of Large White pigs, and we found a higher percentage of type IIB fibers [13,14,15]. Thus, it could be speculated that *FIT1* gene may participate in the type I to type II fiber change during postnatal muscle development. Besides the skeletal muscle fiber transitions, other factors may influence meat quality as well, such as body weight, lipid content, PH, and tenderness [16,17]. Intramuscular triglyceride content is an important determinant of pork meat flavor [2]. An insertion/deletion mutation in porcine *FIT1* gene coding region was highly associated with a series of fat traits [3]. Therefore, focus on the regulation of porcine *FIT1* gene in skeletal muscle is very important in agriculture.

The progressive deletions showed that the fragment from −397 to +3 bp maintained almost the full activity exclusively in C2C12 myotubes while lost the activity in C2C12 myoblast cells, C3H10T 1/2 fibroblast cells as well as PK15 cells. This finding demonstrates the crucial role of this promoter sequence in activating porcine *FIT1* promoter activity during C2C12 myotube formation. On the other hand, the promoter activity of porcine *FIT1* gene was detected exclusively in C2C12 myotubes rather than in porcine PK15 cells, which may suggest the lack of myotube environment required for transcription initiation in this cell type. Alternatively, it may be as a consequence of absence of the mouse-specific cofactor in PK15 cells. Despite the fact that additional experiments are required to confirm this hypothesis in porcine myoblast cells, it is likely that muscle-specific regulatory elements reside in this region and are thus implicated in next step searching for myogenic transcription factors binding to this region. In addition, as the RNA samples used for expression pattern analysis are from large White pigs and the templates to isolate the proximal promoter from Meishan pigs, we sequenced ~1 kb of the promoter sequences from two breeds to confirm whether there is any difference in promoter sequences between large White pig and Meishan pig. Our results showed that only one SNP was detected in this region, which was located at −880 bp upstream the coding region. However, our work demonstrated that this region from −1037 to −875 bp did not contain elements required for transcription activity. Therefore, we can conclude that the difference is not the result of the pig breed difference.

We next sought the specific sequence that could be accountable for the myogenic activation of *FIT1* transcription during C2C12 differentiation. Interestingly, two E-box elements were present in fragment from −397 to +3 bp and by aligning the proximal promoter sequence among pig, mouse, rat, and human were found conserved across these species. E-box is typically bound by MyoD and is critical to the induction of plenty of myogenic genes such as *MCK* [18]. By removal of the two E-box and adjacent regions from the *FIT1* promoter, we report an essential role of the region from −245 to +3 bp in modulating the strong transcriptional induction of the porcine *FIT1* gene during C2C12 myotube formation. This finding suggests that there may exist a myogenic enhancer. Furthermore, by mutating the specific nucleotides in the E-box1 and E-box2 sequence, we first report the base alterations in either E-box are the key to inducing the strong *FIT1* promoter activity during the myotube formation. We concluded that the proximal consensus E-box1 sequence (CACCTG) plays the dominant role in activating *FIT1* transcription in C2C12 myotubes. Interestingly, our results also indicate that the non-canonical E-box2 sequence (CAGCTG) in this region remained functional in part, as the luciferase activity was adequate to be modestly reduced by this site mutation.

Given the previous finding that E-box can be bound by MyoD homodimers or MyoD-E2A heterodimers [8], as well as our bioinformatic prediction, it is of special interest to evaluate the importance of MyoD in regulating the promoter activity of *FIT1*. Results show that over-expression of MyoD1 dramatically enhanced the promoter activity of *FIT1* while its inhibition eliminated most of the activity, indicating the essential role of MyoD1 in *FIT1* transcription induction. These findings suggest MyoD is a transcription activator of *FIT1* gene, consistent with the previous report [10]. Intriguingly, we noticed that in the region adjacent to the E-box1 element exists the E2A (E12 and E47) binding site whereas G-quadruplex DNA (such as SP1 binding site) with a tendency to form a variety of tetrahelical structures [19] neighbors the E-box2 element. Both E-box and tetraplex DNA could be bound by MyoD [20], however, transcriptional initiation is activated through MyoD-E12/E47 complex binding to the canonical E-box rather than MyoD homodimers to tetraplex DNA structures in myogenic gene promoters or enhancers [8]. Our findings that MyoD induces myogenic transcription of *FIT1* gene through specific binding to the consensus E-box 1 (CACCTG) support this. It might to some extent demonstrate the previous finding that MyoD-E2A heterodimers form the complex with the canonical E-box sequence more tightly than MyoD homodimers with tetraplex/E-box complexes [8]. This may also partly explain the differential role of E-box1 and E-box2 in regulating *FIT1* promoter activity. Moreover, our result that mutation of non-canonical E-box2 simply partly eliminated the promoter activity was in agreement with the finding of Shklover *et al.* [21] that a firefly luciferase reporter gene expression driven by MyoD and E-box, was enhanced by DNA quadruplexes. Interestingly, one of our findings that MyoD1 binds to the canonical E-box site of the *FIT1* promoter may offer indirect evidence to the report of Winokur *et al.* [22] that many FSHD dysregulated genes are direct targets of MyoD.

**Figure 5:**
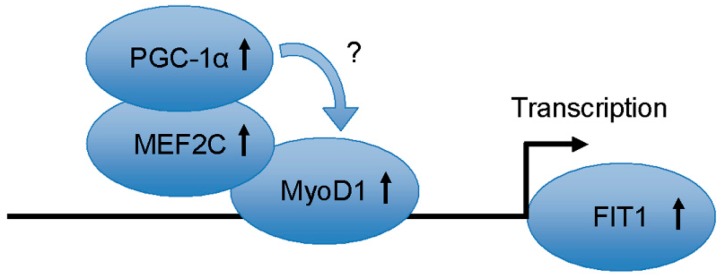
A proposed model to elucidate the activation of FIT1 by PGC-1α via MyoD during myogenesis.

In addition, Mormeneo *et al.* showed that overexpression of PGC-1α activated the expression of *FIT1* and protected the muscle from atrophying as well as increasing the triglyceride level and glucose transport in cultured human skeletal muscle cells [23]. Combined with our findings that *FIT1* gene could be activated by myogenic transcription factor MyoD1, it is interesting to explore the relations between PGC-1α and MyoD. It is previously known that PGC-1α contains a key functional domain used for interacting directly with MEF2 [24], and MEF2C is a direct transcriptional target of MyoD [25]. We therefore propose that induction of PGC-1α on *FIT1* gene may require the additional cooperative involvement of MEF2C and MyoD. It is not yet clear whether FIT1, as a target of PGC-1α, is activated by the direct interaction of PGC-1α and MyoD. However, at least it is an alternative to mediate this activation of *FIT1* by PGC-1α through cooperating with MyoD, with MEF2C as a partner. The possible model is shown in Figure 5. Another study demonstrated that MyoD could directly cooperate with MEF2 protein family members to initiate the myogenic gene expression [26,27]. It is also reported that PGC-1α coactivated MEF2 to stimulate the level of the muscle genes coupled with development and glucose metabolism [28]. In combination with the fact that MEF2C has certain effects on *FIT1* promoter activity, the proposed pathway involved in the activation of *FIT1* via the interaction between PGC-1α and MyoD could therefore be reasonable. Although more evidence is still needed to prove whether PGC-1α is a coactivator of MyoD, this hypothesis could be helpful to further study the interactions of transcription factors with *FIT1* promoter during muscle development.

Taken together, for the first time we identify an enhancer site encompassing two E-box motifs (especially the canonical E-box1) that govern *FIT1* promoter activity during C2C12 differentiation, and propose this to some extent explains the myogenic expression of *FIT1* gene across different mammals. We also demonstrate that porcine *FIT1* promoter is transactivated by MyoD1 that binds to the canonical E-box1 site in C2C12 myotubes. This is the first demonstration of the interaction between MyoD1 and *FIT1* promoter during myogenesis and MyoD1 is the first transcription factor recognized to function in *FIT1* promoter region. We therefore extend the previous work on mechanisms of *FIT1* gene in skeletal muscle regulation except for its role in TG accumulation [1], as well as provide new directions for future study on the roles of *FIT1* gene in muscular dystrophy through targeting the E-box sequence.

## 4. Experimental Section

### 4.1. Isolation of the Porcine FIT1 5′-Flanking Region

Genomic DNA of Chinese Meishan pig was acquired from the skeletal muscle following the instructions of the genomic DNA isolation kit (Tiangen, Beijing, China). The porcine *FIT1* 5′-flanking region was obtained by alignment with its cDNA sequence (Accession number FJ393218) to the genomic DNA sequence. Primers used to amplify the fragment including the promoter and 5′ untranslated region were listed in Table S1. The potential transcription factor binding sites and the predicted transcription start site were analyzed employing Neural Network Promoter Prediction and TESS online program (Available online: http://www.cbil.upenn.edu/cgi-bin/tess/tess).

### 4.2. RNA Extraction, cDNA Synthesis and qRT-PCR

Total RNAs were extracted from skeletal muscle of Large White pigs at embryonic, 2-, 4-, 6-month of age respectively, using the RNA extraction kit (Omega, Norcross, GA, USA). cDNAs were synthesized according to the instructions of the kit (Invitrogen, Shanghai, China). Real time RT-PCR was performed on the CFX96 Real-time System (Bio-Rad, Hercules, CA, USA) to analyze *FIT1* expression with 1 µL cDNA, 10 µL SYBR Green I Super-mix (TOYOBO, Osaka, Japan), and 10 nM specific primers in a total volume of 20 µL. *HPRT* (hypoxanthine guanine phosphoribosyl transferase) gene was also amplified for internal standard and primers were shown in Table S1. PCR amplifying conditions were listed: 1 cycle of 95 °C for 2 min, 40 cycles of 95 °C for 15 s, 57 °C for 15 s, 72 °C for 20 s, and the relative level of *FIT1* gene was calculated by 2^−∆∆*C*t^ [29].

### 4.3. Luciferase Vector Construction, Cell Culture and Transfection

Porcine PK15 cells, C3H10T 1/2 fibroblast cells, and C2C12 myoblast (All from China Center for Type Culture Collection (CCTCC), Wuhan, China) cultures were fed in Dulbecco’s modified Eagle’s medium (DMEM, Gibco, Carlsbad, CA, USA) with 10% fetal bovine serum (FBS, Gibco) at 37 °C with 5% CO_2_. For induction of differentiation, DMEM medium with 2% horse serum was added to C2C12 myoblast cells for 72 h. A series of progressive 5′-deletion fragments were amplified by PCR, then ligated into pGL3-basic vectors (Promega, Madison, WI, USA) and confirmed by sequencing (Sangon, Shanghai, China). The MyoD1 eukaryotic expression vector was a gift from Wangjun Wu (Nanjing Agricultural University, Nanjing, China). Cells were plated in 24-well plates and transiently transfected with plasmids when they reached ~80% confluence according to the protocol of Lipofectamine 2000 (Invitrogen).

### 4.4. Assay of the Luciferase Activity

The transfected cells were lysed and the supernatants were obtained by centrifugation after transfection for 24 h. The transcriptional activity was then measured with the dual-luciferase reporter assay kit (Promega) in triplicates for each treatment in luminometer (GloMax 96 Microplate, Promega, Madison, WI, USA). A pRL-TK (50 ng) plasmid was co-transfected with deletion constructs to correct for the transfection efficiency.

### 4.5. siRNA Interference

The siRNA against MyoD1 was designed as described previously [30] and synthesized with the control siRNA (GenePharma Co., Ltd., Shanghai, China). The siRNA sequences were shown in Table S1.

### 4.6. Chromatin Immunoprecipitation (ChIP) Assay

ChIP assay was conducted using chromatin immunoprecipitation kit (Millipore, Billerica, MA, USA). Specifically, C2C12 myoblasts were differentiated for 3 days and fixed with 1% formaldehyde for 10 min at 25 °C followed by glycine neutralization for 5 min. Prior to sonication, cell lysates were rinsed by ice-cold PBS, and then fragmented to the size between 200 and 750 bp. The ChIP Dilution Buffer containing Protease Inhibitor Cocktail II was mixed with the sonicated chromatin above, followed by overnight incubation with anti-MyoD1 antibody (Abcam, Shanghai, China) at 4 °C with rotation. Precipitated by Protein G Agarose, the resulting complex was washed in the cold buffers as indicated by the instructions (Millipore). Lastly, Elution Buffer (20% SDS, 1 M NaHCO_3_) was employed to elute the beads, and 5 M NaCl was to reverse the cross-linked DNA-protein complex at 65 °C overnight. Post-treatment by proteinase K at 45 °C for 1 h, the expected DNA targets were obtained by PCR using the following protocol: 3 min at 94 °C; 26 cycles of 94 °C for 20 s, 66 °C for 30 s, and 72 °C for 8 s. ChIP-qPCR was performed to quantitate relative enrichments of the regions containing E-box in the FIT1 promoter. Primers used are listed in Table S1.

### 4.7. Statistical Analysis

Means ± standard deviation (SD) was used to indicate the results. Comparisons between two groups were made by student’s *t* test and one-way ANOVA.

## Supplementary Materials

Supplementary materials can be found at http://www.mdpi.com/1422-0067/16/10/25014/s1.
