# Supplementary material for: MyoD Is a Novel Activator of Porcine FIT1 Gene by Interacting with the Canonical E-Box Element during Myogenesis
Source: Int J Mol Sci. 2015 Oct 20;16(10):25014–30. doi: 10.3390/ijms161025014 (PMC4632787; doi:10.3390/ijms161025014)
Supplement: Supplementary file 1 [file ijms-16-25014-s001.pdf]

Supplementary Information

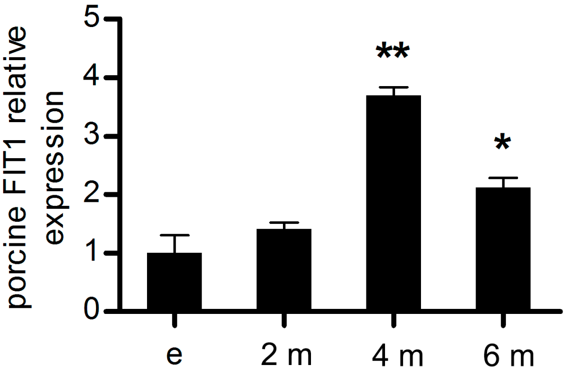

(A)

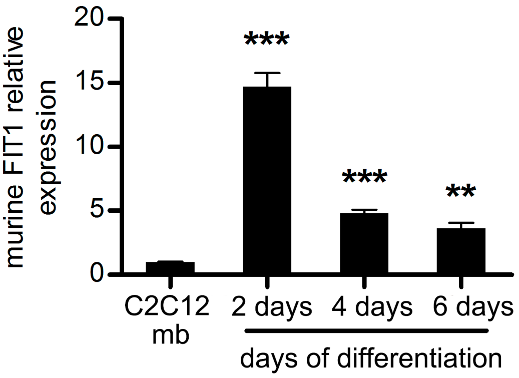

(B)

Figure S1. FIT1 mRNA expression profiles during development of the longissimus dorsi muscle in Large White pigs (**A**) and over the differentiation of C2C12 myoblasts (**B**). The relative expression (means ± SD, $n = 3$) was normalized to the level of the embryonic stage (**A**) and of the non-differentiated C2C12 myoblast cultures (**B**) with HPRT as the internal control, respectively. * $p < 0.05$; ** $p < 0.01$; *** $p < 0.001$ by ANOVA. e, embryonic stage; 2 m, 2 months of age; 4 m, 4 months of age; 6 m, 6 months of age; mb, myoblast.

Table S1. Primers used in plasmid construction and qRT-PCR.

Primer	Sequence (5′→3′)
pGL3-FIT1(−1037/+3)-F	CGACGCGTGGAGTGGGATGGATGG
pGL3-FIT1(−875/+3)-F	CGACGCGTTGGGTCACCATGCTGT
pGL3-FIT1(−573/+3)-F	CGACGCGTTCGGCATTCTCACCACT
pGL3-FIT1(−397/+3)-F	CGACGCGTGGTGAGGTGGGGATGA
pGL3-FIT1(−123/+3)-F	CGACGCGTGCCAACACAGCTCTGC
pGL3-FIT1-R(3′)	GGAAGATCTCATGTTCCCTCCCCTC
pGL3-mut(−870/−245)-F	GGGGTACCCACCATGCTGTACAGT
pGL3-mut(−384/−245)-F	GGGGTACCTGACTTTGACCAAACAGG
pGL3-FIT1-mut-R(3′)	GAAGATCTGTATCCTTCCCTCAGTCC
qPCR-FIT1-F(pig)	CAACCCTCGGACCAT
qPCR-FIT1-R(pig)	GTAGCCAGGAACACCA
qPCR-HPRT-F(pig)	GGTCAAGCAGCATAATCCAAAG
qPCR-HPRT-R(pig)	CAAGGGCATAGCCTACCACAA
qPCR-FIT1-F(mouse)	CGATGAGTAGGAAGGCAC
qPCR-FIT1-R(mouse)	CACGGCAACTTCTTCAAC
qPCR-HPRT-F(mouse)	GCAAACTTTGCTTTCCCTGG
qPCR-HPRT-R(mouse)	GCTTTGTATTTGGCTTTTCC
siRNA of MyoD1-F	CCAAUGCGAUUUAUCAGGUGCUUUGTT
siRNA of MyoD1-R	CAAAGCACCUGAUAAAUCGCAUUGGTT
E1-F	GCCAACACAGCTCTGC
E1-R	CATGTTCCCTCCCCTC
E2-F	CTATGTCAGGACACACTTCCA
E2-R	GGGCAGAGCGCTGTTG
NC1-F	CCTCTGCCTTACTGTACTTTG

Table S1. *Cont.*

Primer	Sequence (5′→3′)
NC1-R	GATGACCACTGCTGCCAA
NC2-F	TGGGTGCGGCAGTAGG
NC2-R	GCGCATTGAGCGGGAT
